# Effect of microwave sterilization on maturation time and quality of low‐salt sufu

**DOI:** 10.1002/fsn3.1346

**Published:** 2019-12-12

**Authors:** Xuejing Fan, Xuepeng Lv, Li Meng, Mingzhi Ai, Chunqiu Li, Fei Teng, Zhen Feng

**Affiliations:** ^1^ Key Laboratory of Dairy Science Ministry of Education College of Food Science Northeast Agricultural University Harbin Heilongjiang China

**Keywords:** low‐salt, maturation time, microwave sterilization, qualitative property, sufu

## Abstract

The objective of this study was to reduce the microorganism number and salt content in pehtze by microwave sterilization. The maturation time and quality of low‐salt sufu were evaluated. The microorganism inactivation rate, moisture content and water activity of the pehtze, which was used for the growth of the starter culture, showed that 4,250 W for 30 s was suitable for the preparation of low‐salt sufu. With regard to the physicochemical properties of sufu, 120‐day sufu samples obtained by traditional high‐salt (14%) fermentation and 75‐day sufu samples obtained by low‐salt (4%) fermentation met the standard requirements. With regard to the sensory characteristics of sufu, the taste and after taste scores of 75‐day low‐salt sufu samples were significantly higher than those of 120‐day high‐salt sufu samples (*p* < .05).The overall acceptance score of low‐salt sufu samples also was higher than that of high‐salt sufu samples. The contents of free amino acids and the profiles of typical flavor compounds partly explained the sensory quality and shorter ripening time of sufu manufactured. The total biogenic amine contents were reduced by 46%.

## Graphical abstract

In this work, we reduce the microorganism numbers in pehtze and to prepare low‐salt sufu using microwave sterilization. Furthermore, the qualitative properties of low‐salt sufu prepared by this approach and traditionally fermented high‐salt sufu were evaluated. The overall objective was to provide the theoretical foundation for the preparation of low‐salt Kedong sufu.

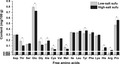

## 1 INTRODUCTION

Sufu is a traditionally fermented soybean product in China. It is a soft, creamy, cheese‐like product made from cubes of soybean curd by microbial action. Sufu is similar to cheese in terms of its processing and ripening mechanism, and sufu can be used in the same way as cheese in food preparation. Thus, sufu is regarded as the “Chinese cheese” (Steinkraus, 1997). It has been widely consumed by the Chinese people as an appetizer for more than 1,000 years. Sufu can be divided into four types based on the manufacturing process, namely mold‐fermented sufu, enzymatically ripened sufu, naturally fermented sufu and bacteria‐fermented sufu. Sufu is usually prepared by the addition of 10%–15% salt, which is required for its preservation. However, high levels of salt intake can cause various health problems, such as hypertension, osteoporosis, and gastricism, which are typically observed in many societies (Mcnaughton, Ball, Mishra, & Crawford, 2008). The salt content of foods, especially traditionally fermented food products, has recently been of concern to consumers’ (Lee, Ahn, Jo, Yook, & Byun, 2010). In addition, a high salt concentration can inhibit the growth of the starter culture and its enzymatic activity, thereby prolonging the maturation time of sufu (Guan et al., 2013; Ma, Cheng, Yin, Wang, & Li, 2013).

Different methods have been employed to reduce the salt content in traditionally fermented foods, including the use of salt substitutes or other seasonings that contain additives such as ethanol, potassium chloride and calcium chloride. Other investigators have altered the fermentation conditions in the hopes of lowering the salt content (Chiou, Ferng, & Beuchat, 1999; Dos Santos et al., 2015). However, low‐salt fermented foods are not palatable, and the growth of pathogenic bacteria can reduce the shelf life. The gamma irradiation method has also been used to prepare low‐salt fermented food products (Jo et al., 2004; Kim, Kim, Ahn, Park, & Byun, 2005; Song et al., 2010). However, irradiation causes lipid oxidation, thereby altering the relative ester contents and producing an off‐odor (Groninger, Tappel, & Knapp, 2010; Wang et al., 2010). Microwave sterilization can reduce the microorganism number, which ensures food safety and extends the shelf life of food (Chen et al., 2016). Furthermore, microwave sterilization does not affect the quality of food, especially its texture, color and flavor (Guo, Sun, Cheng, & Han, 2017; Vega‐Miranda, Santiesteban‐López, López‐Malo, & Sosa‐Moralesa, 2012). Kedong sufu is a typical bacteria‐fermented sufu in China (Feng et al., 2014). Its salt content is 14%. Reducing its salt content without altering any important characteristics would be very advantageous. However, there is no study reporting the preparation of low‐salt sufu using microwave sterilization.

The objective of this study was to reduce the microorganism numbers in pehtze and to prepare low‐salt sufu using microwave sterilization. Furthermore, the qualitative properties of low‐salt sufu prepared by this approach and traditionally fermented high‐salt sufu were evaluated. The overall objective was to provide the theoretical foundation for the preparation of low‐salt Kedong sufu.

## 2 MATERIALS AND METHODS

### 2.1 Preparation of traditionally fermented high‐salt sufu and low‐salt sufu

The pilot study of Kedong sufu was carried out in a sufu‐making plant located in Kedong (Kedong, Heilongjiang, China). The pehtze was also from the sufu‐making plant. The starter culture suspension was sprayed onto the surface of the pehtze evenly and the production of sufu was carried out according to Feng and colleagues (Feng et al., 2014, 2016). The inoculated pehtzes were placed in plastic trays so that they were evenly spaced. The loaded trays were stacked in an incubation room with controlled temperature (30°C) and relative humidity (90%). After 7 days, pehtzes were placed in individual wide‐mouth glass bottles with a capacity of 300 ml. Dressing mixture was added to the glass bottles. The filled bottles were incubated at 25°C for fermentation. Two different batches of sufu were prepared, namely sufu prepared from pehtze and microwave sterilized (salt content 4%) and sufu prepared from pehtze and not microwave sterilized (salt content 14%).

### 2.2 Optimization of microwave sterilization parameters

**Figure 1:**
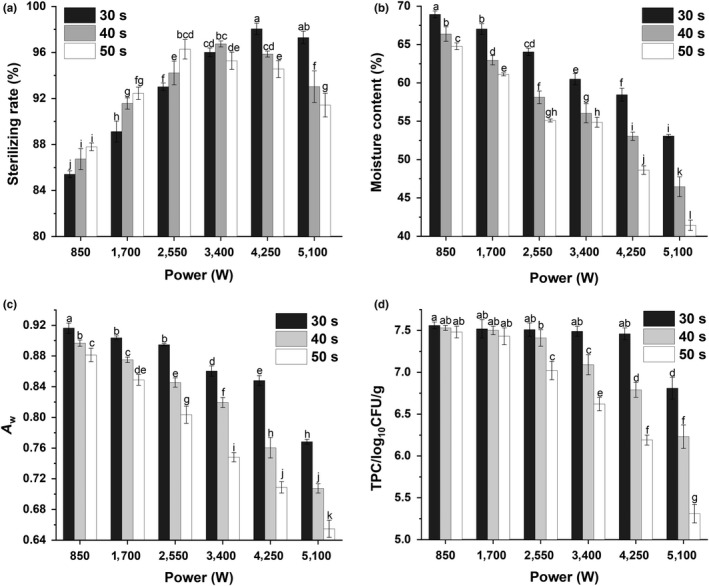
The effects of microwave power and time on the (a) microorganism inactivation rate, (b) moisture, (c) water activity (*a*_w_), and (d) growth of the starter culture in pehtze (different letter showed significant difference, *p* < .05)

A microwave sterilizer (WB7.5E, Nanjing Keller Electric Microwave Equipment Co., Ltd., China) was used to treat the pehtze. Figure 1(a) shows the microwave power and time. The microwave sterilization parameters were selected based on the microorganism inactivation rate, moisture content, water activity (*a*_w_) and growth of the starter culture in pehtze.

For microbiological analysis, samples (25 g) were transferred to individual sterile plastic bags and homogenized in 225 ml of sterile saline solution. Appropriate decimal dilutions of the homogenates were spread on plates of tryptic soy broth (TSB) agar (Becton, Dickinson and Co., Sparks, MD, USA) and incubated at 30°C for 48–72 hrs. For moisture and water activity analyses, the moisture content was determined by removing the moisture at 105°C and then calculating the weight loss as a percentage of the initial weight. The *a*_w_ was measured with a Manual Water Activity Series 3TE Meter (AquaLab, Washington, USA) at 37°C with an accuracy of ± 0.003. Each sample underwent three measurements.

### 2.3 Analysis of physicochemical and sensory characteristics of sufu

The physicochemical characteristics of the sufu samples were determined in accordance with a national standard method (SB/T10170 2007). The water‐soluble protein content was determined by the trace Kjeldahl method.

With descriptive analysis, the sensory characteristics were evaluated by eight panelists from the Quality Control Department of Kedong Sufu Co., Ltd, 4 males and 4 females between 20 and 40 years of age, who were superior sufu quality inspectors and were also regular sufu consumers. All panelists were familiar with basic sensory evaluation techniques. Before sensory evaluation, they participated in briefing sections to familiarize themselves with the specific vocabulary used to describe the sufu attributes and they were trained for 20 days on the standard recommendations (ISO, 1993) with a focus on sufu characteristics. The training course comprised two steps. In a first step, the panelists were handed samples of different ages and were asked to describe the visual and orthonasal impression. Until the panelists had strong ability of sensory memory and different sensory levels, and they could distinguish the fermentation prophase, the fermentation metaphase and the end of fermentation of sufu by sensory analysis. Furthermore, panelists could associate fermentation process with microorganisms known to produce a certain sensory effect through sensory analysis. During the second step, all mentioned attributes were reviewed as part of a group discussion. The samples were labeled with three‐digit random numbers. Ranking of samples was conducted first where one piece each of eleven samples, placed in small cups, was all served simultaneously. The serving order was randomized and panelists were given verbal instructions to start to taste the first sample from an indicating mark on the disposable serving plate then proceed tasting samples clockwise one by one. In addition, a very neutral noncarbonated mineral water and unsalted crackers were used to clean the palate between samples evaluation. The eleven samples were ranked from most to least preferred using the provided scale. The flavor, residual intensity flavor, texture, odor, appearance and overall impression of the sufu were evaluated on a 5‐point intensity scale (1 = bad, 2 = slightly bad, 3 = neutral, 4 = good, 5 = excellent). These criteria were defined as follows: (i) flavor: the association of the flavor with the typical characteristics of bacteria‐fermented sufu; (ii) residual intensity flavor: the intensity of the flavor during mastication and the duration of the flavor; (iii) texture: the degree of exquisite, soft and uniform texture; (iv) odor: the intensity of the smell and the specificity of the smell for this type of product, which should have had a slight aroma of an ester compound; and (v) appearance: the external visual impression. After a brief recess to allow panelists to rest, the same panelists were presented with the same test again and repeated the same test at least three times.

### 2.4 Analysis of free amino acids and typical flavor components in sufu

The contents of free amino acid were measured with a Beckman 6,300 High Performance Amino Acid Analyzer (Beckman Instruments Ltd., High Wycombe, UK) fitted with a 120 × 4 mm cation‐exchange column (Na^+^ form). The procedure was performed as described previously (Feng, Chen, Li, & Ren, & Dan., 2013). The typical flavor components of the sufu were extracted by the SPME‐GC‐MS method as described previously (Sidira, Kandylis, Kanellaki, & Kourkoutas, 2015) with minor modifications. The sufu samples (25 g) were placed in a 50‐mL headspace vial. The samples were stirred with a thermostatic water bath set at 65°C for 30 mins to accelerate the equilibrium of the headspace volatile substances between the headspace and the sample matrix. Thereafter, the SPME fiber (PDMS/DVB/CARB, Supelco, USA) was inserted into the headspace bottle for absorption for 40 mins. The fiber was then inserted into the injection port of the gas chromatograph rapidly and desorbed at 250°C for 2.5 mins under splitless injection mode. The analysis of the volatile compounds was performed with an Agilent 6890–5973 Gas Chromatography‐Mass Spectrometer.

### 2.5 Analysis of biogenic amines

The analysis of the biogenic amines in the sufu samples was performed by high‐performance liquid chromatography (HPLC; Agilent 1,100, Hewlett Packard series‐1100, Santa Clara, CA, USA). The procedure was performed as described previously (Guan et al., 2013). The biogenic amine standards were purchased from Sigma (Sigma‐Aldrich Chemie GmbH).

### 2.6 Statistical analysis

Each experiment was performed at least three times. Data were analyzed using the SPSS Statistical Package (version 22.00 for Windows, 2010), and the differences among the mean values were processed by the least‐significant difference multiple range test. Significance was defined at *p* < .05.

## 3 RESULTS

### 3.1 Effects of microwave power and time on the microorganism inactivation rate, moisture and water activity in pehtze

The effects of microwave power and time on the microorganism inactivation rate, moisture, water activity and growth of the starter culture in pehtze are shown in Figure 1(a)‐(d). The results showed that the microorganism inactivation rate increased significantly (*p* < .05) with increasing microwave power and time when the microwave power was ≤ 4,250 W. The microorganism inactivation rate (98.05%) was the highest at 4,250 W for 30 s. However, the microorganism inactivation rate decreased to 95.85% and 94.55% when pehtze was microwave sterilized at 4,250 W for 40 and 50 s, respectively, and it decreased to 97.29%, 93.02%, and 91.42% when pehtze was microwave sterilized at 5,100 W for 30, 40, and 50 s, respectively.

The moisture content decreased significantly (*p* < .05) from 68.93% to 41.42% with increasing microwave power and time. The moisture content was 58.45%, 53.03%, and 48.62% when pehtze was microwave sterilized at 4,250 W for 30, 40, and 50 s, respectively, and it was 53.07%, 46.45%, and 41.42% when pehtze was microwave sterilized at 5,100 W for 30, 40, and 50 s, respectively.

The *a*_w_ of pehtze was within the range of 0.65–0.92. The *a*_w_ of pehtze treated for 30 s (0.768–0.916) was higher than that of pehtze treated for 40 s (0.707–0.897) and 50 s (0.654–0.881). In addition, the *a*_w_ of pehtze treated at 2,550 W for 50 s (0.803), 3,400 W for 40 s (0.819), and 5,100 W for 30 s (0.768) were lower than that of pehtze treated at 4,250 W for 30 s (0.848).

Figure 1(d) shows the effects of microwave power and time on the growth of the starter culture in pehtze. Figure 1(c) and (d) shows the relationship between the growth of the starter culture and the *a*_w_ of raw materials treated by microwave sterilization. The results showed that the colony count decreased significantly (*p* < .05) from 7.56 log_10_CFU/g to 5.31 log_10_CFU/g with increasing microwave power and time. Furthermore, the colony count decreased with decreasing *a*_w_. However, there was no remarkable difference (*p* > .05) in the colony count between the pehtze microwave sterilized at 4,250 W for 30 s (7.46 log_10_CFU/g) and the pehtze microwave sterilized at 850 W for 30 s (7.56 log_10_CFU/g). Because the *a*_w_ was 0.848 when pehtze was treated at 4,250 W for 30 s, the colony count reached 7.46 log_10_CFU/g.

Based on the results of the microorganism inactivation rate, moisture content, water activity and growth starter culture in pehtze, a microwave power and time of 4,250 W and 30 s exhibited the highest microorganism inactivation rate as well as the most suitable moisture content and water activity for the growth of the starter culture. The optimal microwave sterilization conditions were selected to prepare low‐salt sufu.

### 3.2 Physicochemical properties of low‐salt sufu and traditionally fermented high‐salt sufu

The contents of total acid, amino acid nitrogen, moisture and water‐soluble proteins are important standards for determining the ripeness of sufu (SB/T10170 2007). The results are shown in Table 1. Although the total acid content was significantly higher in traditionally fermented high‐salt sufu at 120 days of ripening than that in low‐salt sufu at 75 days of ripening, the levels of amino acid nitrogen and water‐soluble proteins were lower than those of low‐salt sufu. Water‐soluble proteins and amino acid nitrogen were significantly affected by the salt content. After 75 days of ripening, the levels of total acid, amino acid nitrogen and water‐soluble proteins in the low‐salt sufu met the national standards (SB/T10170 2007). Traditionally fermented high‐salt sufu met the standards after 120 days of ripening.

**Table 1:** The contents of total acid, moisture, amino acid nitrogen, and water soluble proteins in low‐salt sufu and high‐salt sufu during ripening (mean ± *SD*)

Sample	Time (days)	Total acidity (g/100 g)	Moisture (g/100 g)	Amino acid nitrogen (g/100 g)	Water soluble proteins (g/ 100g)
Low‐salt sufu	30	0.56 ± 0.01^de^	59.12 ± 2.26^a^	0.22 ± 0.01^ef^	1.43 ± 0.03^h^
	45	0.61 ± 0.02^d^	60.24 ± 1.27^a^	0.34 ± 0.04^d^	2.49 ± 0.06^ef^
	60	0.72 ± 0.04^bc^	60.73 ± 1.84^a^	0.41 ± 0.02^b^	3.28 ± 0.07^c^
	75	0.78 ± 0.03^b^	61.27 ± 1.19^a^	0.48 ± 0.03^a^	3.77 ± 0.04^a^
High‐salt sufu	30	0.38 ± 0.01^f^	60.94 ± 1.28^a^	0.18 ± 0.01^f^	1.15 ± 0.07^i^
	45	0.42 ± 0.02^f^	61.13 ± 2.08^a^	0.21 ± 0.01^f^	1.41 ± 0.07^h^
	60	0.50 ± 0.05^e^	61.37 ± 1.98^a^	0.26 ± 0.01^e^	1.94 ± 0.08^g^
	75	0.59 ± 0.06^d^	61.52 ± 2.13^a^	0.33 ± 0.02^d^	2.37 ± 0.10^f^
	90	0.69 ± 0.08^c^	61.84 ± 1.87^a^	0.36 ± 0.04^cd^	2.61 ± 0.09^e^
	105	0.76 ± 0.04^b^	61.91 ± 2.34^a^	0.39 ± 0.05^bc^	3.15 ± 0.10^d^
	120	0.85 ± 0.02^a^	62.04 ± 2.16^a^	0.43 ± 0.03^b^	3.52 ± 0.08^b^
SB/T10170−2007		≤1.2	≤72	≥0.42	≥3.5

Note

All the data expressed in the unit of g/100 g of sufu. Mean values with different superscripts letters in the same column are significantly different at *p* < .05 by SPSS and the LSD test.

### 3.3 Sensory evaluation of low‐salt sufu and traditionally fermented high‐salt sufu

The results of the sensory characteristics analysis for low‐salt sufu and traditionally fermented high‐salt sufu are shown in Table 2. There were significant differences in the sensory characteristics at different fermentation times. All sensory characteristic scores for low‐salt sufu reached a maximum after 75 days of fermentation. The taste and after taste scores of low‐salt sufu after 75 days of fermentation were higher than those of the high‐salt sufu after 120 days of fermentation (*p* < .05). Although the odor, texture and color scores of low‐salt sufu were higher than those of high‐salt sufu, there was no significant difference (*p* > .05). In addition, the overall acceptance score of the low‐salt sufu after 75 days of fermentation was higher than that of the high‐salt sufu after 120 days of fermentation.

**Table 2:** The sensory evaluation of low‐salt sufu and high‐salt sufu during ripening (mean ± *SD*)

Sample	Time (days)	Odor	Taste	After taste	Texture	Color	Overall acceptance
Low‐salt sufu	30	1.4 ± 0.2^g^	1.4 ± 0.3^fg^	1.5 ± 0.1^e^	1.8 ± 0.2^f^	2.5 ± 0.2^fg^	2.1 ± 0.1^ef^
	45	2.3 ± 0.3^e^	2.4 ± 0.1^e^	2.9 ± 0.2^cd^	2.7 ± 0.1^d^	2.9 ± 0.4^de^	3.2 ± 0.2^cd^
	60	3.6 ± 0.2^d^	3.9 ± 0.2^b^	4.0 ± 0.2^b^	3.2 ± 0.3^c^	3.8 ± 0.1^bc^	4.4 ± 0.3^ab^
	75	4.8 ± 0.2^a^	4.9 ± 0.1^a^	4.9 ± 0.1^a^	4.6 ± 0.2^a^	4.3 ± 0.2^a^	4.8 ± 0.1^a^
High‐salt sufu	30	1.0 ± 0.3^h^	1.1 ± 0.1^g^	1.1 ± 0.2^f^	1.3 ± 0.1^g^	1.4 ± 0.1^h^	1.3 ± 0.2^g^
	45	1.8 ± 0.1^f^	1.3 ± 0.2^fg^	1.4 ± 0.2^ef^	2.1 ± 0.2^ef^	1.6 ± 0.2^h^	1.9 ± 0.4^f^
	60	2.4 ± 0.2^e^	1.5 ± 0.1^f^	1.6 ± 0.1^e^	2.3 ± 0.2^e^	2.3 ± 0.1^g^	2.5 ± 0.1^e^
	75	3.5 ± 0.3^d^	2.7 ± 0.3^de^	2.7 ± 0.4^d^	2.9 ± 0.3^cd^	2.8 ± 0.3^ef^	3.0 ± 0.4^d^
	90	4.0 ± 0.1^c^	2.9 ± 0.1^d^	3.2 ± 0.1^c^	3.2 ± 0.2^c^	3.2 ± 0.2^d^	3.5 ± 0.1^c^
	105	4.4 ± 0.2^b^	3.3 ± 0.2^c^	4.0 ± 0.3^b^	3.6 ± 0.4^b^	3.7 ± 0.1^c^	4.2 ± 0.3^b^
	120	4.7 ± 0.1^ab^	3.6 ± 0.2^bc^	4.3 ± 0.2^b^	4.3 ± 0.1^a^	4.1 ± 0.2^ab^	4.7 ± 0.1^a^

Note

Mean values with different superscripts letters in the same column are significantly different at *p* < .05 by SPSS and the LSD test.

### 3.4 Free amino acids profiles of low‐salt sufu and traditionally fermented high‐salt sufu

**Figure 2:**
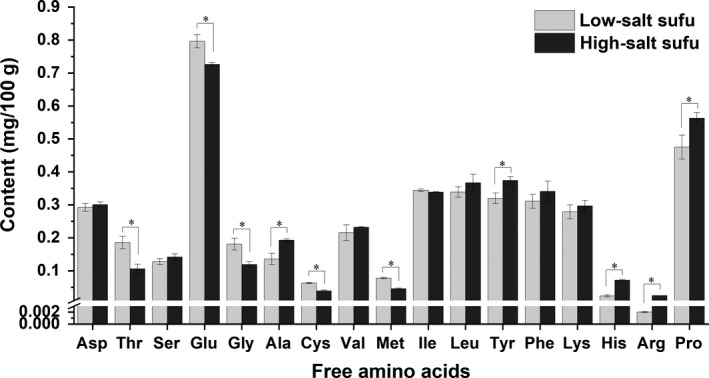
The contents of free amino acids in low‐salt sufu at 75 days of ripening and traditionally fermented high‐salt sufu at 120 days of ripening (*p < .05)

The contents of free amino acids of the low‐salt sufu and traditionally fermented high‐salt sufu were analyzed (Figure 2). Seventeen types of free amino acids were detected in both sufu samples, including seven types of essential amino acids, six types of flavor amino acids and seven types of hydrophobic amino acids. The distribution patterns of the free amino acids were comparable between the high‐salt and low‐salt sufu samples. The content of Glu in both sufu samples was the highest, followed by Pro, Leu, Ile, Asp, Tyr, Phe, Lys, and Val, which were higher than 0.1 g/100 g. The content of Glu in the low‐salt sufu (0.796 g/100 g) after 75 days of ripening was significantly higher than that in the traditionally fermented high‐salt sufu (0.726 g/100 g) after 120 days of ripening. The total contents of Pro, Gly, Ala, Val, Leu, Tyr, and Phe were 2.189 g/100 g in the high‐salt sufu after 120 days of fermentation. However, the content of the amino acids was 1.978 g/100 g in the low‐salt sufu after 75 days of fermentation.

### 3.5 Typical flavor components of low‐salt sufu and traditionally fermented high‐salt sufu

**Figure 3:**
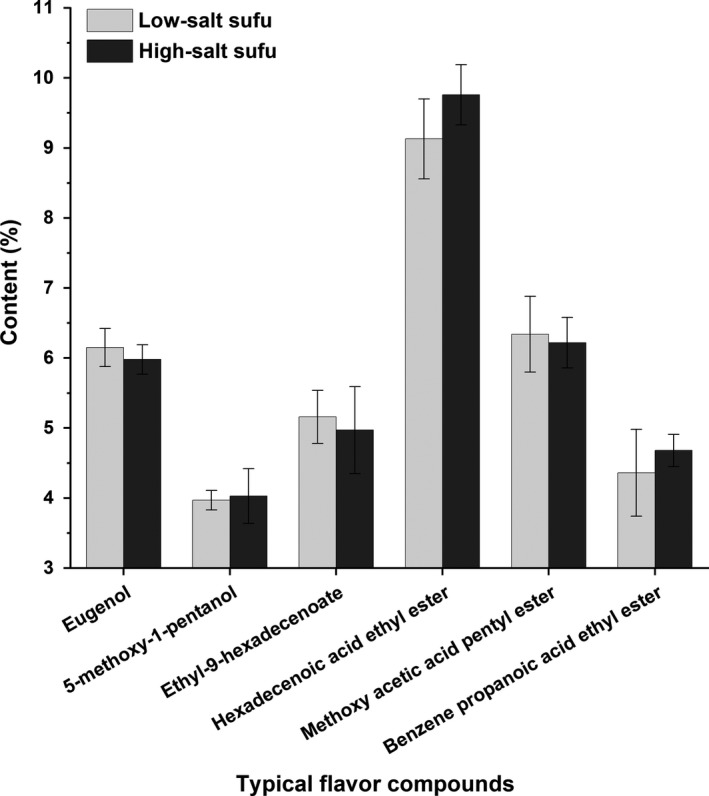
The contents of typical flavor components in low‐salt sufu at 75 days of ripening and traditionally fermented high‐salt sufu at 120 days of ripening (no significant difference)

Flavor is a key attribute that defines the quality and affects the consumer choice of a food product. Figure 3 shows the typical flavor compounds accounted for the proportion of total flavor compounds in the low‐salt sufu after 75 days of ripening and traditionally fermented high‐salt sufu after 120 days of ripening. These flavor constituents were hexadecenoic acid ethyl ester, methoxy acetic acid pentyl ester, benzene propanoic acid ethyl ester, ethyl 9‐hexadecenoate, 5‐methoxy‐1‐pentanol and eugenol, and they contributed greatly to the flavor of sufu. The flavor constituents of the low‐salt sufu and high‐salt sufu were 35.11% and 35.64%, respectively. There was no significant difference in the flavor constituents between the two types of sufu.

### 3.6 Biogenic amines

**Figure 4:**
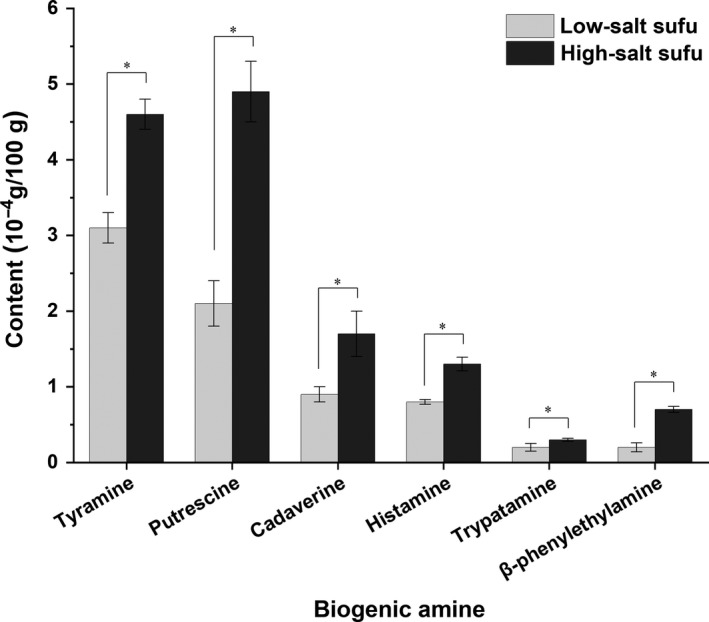
The biogenic amine content in low‐salt sufu at 75 days of ripening and traditionally fermented high‐salt sufu at 120 days of ripening (*p < .05)

The biogenic amine content in the low‐salt sufu at 75 days of ripening and the traditionally fermented high‐salt sufu at 120 days of ripening was determined (Figure 4). Six types of biogenic amines were detected in the sufu samples, including tyramine, putrescine, cadaverine, β‐phenylethylamine, histamine, and tryptamine. The results indicated that contents of tryptamine, β‐phenylethylamine, cadaverine, histamine, and tyramine were lower in the low‐salt sufu than those in the traditionally fermented high‐salt sufu. The biogenic amine content in the low‐salt sufu decreased to 46% compared with that of the high‐salt sufu.

## 4 DISCUSSION

Microwave sterilization can be used to inactivate contaminating microorganisms in food products. Optimizing the microwave sterilization process can be an effective way to inactivate contaminating raw material‐derived microorganisms in traditionally fermented foods as a substitute for the use of high salt concentrations for preservation. Presently, there is no study reporting the use of microwave sterilization in the production of low‐salt sufu. This study highlighted the use of microwave sterilization in the production of low‐salt sufu to inactivate contaminating microorganisms in pehtze as a substitute for the use of high salt concentrations for preservation. The effects of microwave sterilization on maturation time and quality of low‐salt sufu were revealed.

The microorganism inactivation rate of pehtze increased significantly with increasing microwave power and time. Similar results were reported for *Salmonella enteritidis* in potato omelet (Valero, Cejudo, & García‐Gimeno, 2014). The microorganism inactivation rate was highest when pehtze was microwave sterilized at 4,250 W for 30 s, indicating that the microorganism response depends on the microwave power and time (Rougier, Prorot, Chazal, Leveque, & Leprat, 2014). The microorganism inactivation rate was also probably related to the increase in the temperature (Guo et al., 2017; Jamshidi, Seifi, & Kooshan, 2010). However, the temperature at the center might have been influenced by the moisture content. The moisture content decreased with increasing irradiation time under the same microwave power, suggesting that microwave energy can accelerate the diffusion and evaporation of moisture from the inside to the outside (Anwar et al., 2015). Furthermore, the moisture removal rate was affected by temperature, thereby affecting the microorganism inactivation rate (Venkitasamy et al., 2017).

Water activity should also be considered when analyzing the inactivation rate of contaminating microorganisms from raw materials in fermented foods. There is strong relationship between the growth of the starter culture and the *a*_w_ of raw materials treated by microwave sterilization: higher *a*_w_ values associate with faster microbial growth (Elgadir et al., 2011), which might be due to the susceptibility of the microbial membrane during dehydration (Tymczyszyn et al., 2008). The *a*_w_ of all pehtze samples decreased significantly from 850 W for 30 s to 5,100 W for 50 s. The *a*_w_ reflects the mobility of protons in water, with protons, becoming entrapped in a loose protein matrix. Thus, the *a*_w_ of pehtze correlated with its moisture content. The low *a*_w_ might be due to dehydration (Liu, Zhu, Lu, Wei, & Ren, 2015). In addition, the viable counts of the starter culture decreased significantly with decreasing *a*_w_ when the *a*_w_ was ≤ 0.85. Therefore, the lower *a*_w_ of pehtze that was microwave sterilized with a high power and a long time might have suppressed the growth of the starter culture, thereby affecting the maturation time of sufu.

Amino acid nitrogen, free amino acids and water‐soluble proteins are important indices of the quality of sufu. With regard to traditionally fermented soybean products, free amino acids contribute directly to the taste and act as precursors of flavor enhancement (Dajanta, Apichartsrangkoon, Chukeatirote, & Frazier, 2010; Lioe, Wada, Aoki, & Yasuda, 2007; Qin & Ding, 2010). Amino acid nitrogen dictates the degree of aging and optimal taste (Byun et al., 2000). The contents of amino acid nitrogen, free amino acids and water‐soluble proteins from low‐salt sufu samples were significantly higher than those from high‐salt sufu samples. Microwave sterilization could reduce the growth of contaminating microorganisms that competed with the starter culture, which essentially improved the growth of the starter culture. This also increased the secretion of endogenous proteases by the starter culture and controlled the aroma, texture and flavor of the final product (Leroy & Vuyst, 2004). In addition, a high salt concentration can not only inhibit the growth of the starter culture, but also inhibit the activity of endogenous proteases (Xu, Yu, Xue, Xue, & Ren, 2008). These results also indicate that a low salt concentration might have favorable effects on fermentation by reducing the fermentation period, which might be caused by the combined effects of high enzymatic activity (Lopetcharat, Choi, Park, & Daeschel, 2001). A high salt content can increase the hardness of sufu. In other words, a low salt content decreased the hardness and increased the protein concentration and lipid degradation (Xia, Li, Zheng, Ran, & Kan, 2014). Meanwhile, a low salt content in pehtze improved the growth and survival of the starter culture as well as the metabolism of microbes (Mcmahon et al., 2014). Increased proteolysis can affect the texture of the sufu during ripening, whereas salt can affect its bitterness (Engel, Tournier, Salles, Quéré, & Le., 2001). There was no off‐odor detected from the low‐salt sufu samples, which increased consumer acceptance. The total acid content of the low‐salt sufu was lower than that of the high‐salt sufu. In addition, acidity is an important indicator of the shelf life of sufu. After microwave sterilization, the reduced salt content not only shortened the fermentation period of sufu, but also prolonged the shelf life of sufu.

Changes in free amino acids were mainly dominated by proteases that were influenced by the salt content and microbial metabolism (Cui, Zheng, Wu, & Zhou, 2015). In the present study, the total free amino acid contents was higher in low‐salt sufu than that in high‐salt sufu, which might have been attributed to the improved growth of the starter culture and the high protease activity in the low‐salt sufu. The umami taste is attributed to amino acids, particularly Glu, Asp, and Ala (Shu, Kenji, & Osamu, 2011). The Glu content in the low‐salt sufu after 75 days of fermentation was higher than that in the high‐salt sufu after 120 days of fermentation, which might have been be due to hydrolysis by alkaline proteases, as the levels of Asp‐ and Tyr‐cleaving proteases increased (Zhang & Tao, 2009). Sufu samples also had a large amount of Pro, which can contribute to the development of a bitter taste. The presence of Gly, Ala, Val, Leu, Tyr, and Phe in peptides also imparts bitterness as these amino acids are also binding determinants (Zhao, Schieber, & Gänzle, 2016). Hydrophobic amino acids, which possess unique spatial orientations of polar and hydrophobic regions, are also determinants for bitterness (Mi‐Ryung, Kawamura, Ki Myong, & Cherl‐Ho, 2008). However, the contents of these amino acids in the low‐salt sufu were lower than those in the high‐salt sufu. The unbalanced levels of proteolysis and peptide hydrolysis were main reasons for the development of a bitter taste (Engel, Septier, Leconte, Salles, & Le, 2001). A reduced salt content can balance proteolysis. Thus, reducing the salt content could not only shorten the fermentation period, but also increase the contents of total amino acids and umami, thereby decreasing the bitterness. In addition, low‐salt sufu contained essential amino acids at significantly higher levels compared to high‐salt sufu. The sufu produced by microwave sterilization had a higher content of essential amino acids.

Flavor is one of the most important criteria defining food quality and consumer choice and acceptance. Flavor components are formed during ripening (Delgado, González‐Crespo, Cava, García‐Parra, & Ramírez, 2010). Identifying the typical flavor components can help to determine the maturation time and to improve the quality of sufu. The results showed that there was no significant difference in the contents and constitutes of typical flavor components between low‐salt sufu after 75 days of fermentation and traditionally fermented high‐salt sufu after 120 days of fermentation. After microwave sterilization, the low salt concentration in pehtze improved the growth and survival of the starter culture as well as microbial metabolism (Mcmahon et al., 2014). Microorganisms are crucial in the development of flavor components (Molimard & Spinnler, 1996). Thus, microwave sterilization combined with a low salt concentration can accelerate the formation of flavor components during the ripening of low‐salt sufu.

Sufu is produced under an open‐type fermentation environment and stored at ambient temperatures. Sufu has a higher content of biogenic amines compared with other food products (Anastasio et al., 2010; Magwamba, Matsheka, Mpuchane, & Gashe, 2010; Ruiz‐Capillas & Jiménez‐Colmenero, 2004). Bacteria‐generated biogenic amines, namely tyramine, β‐phenylethylamine, and histamine, are readily detectable in fermented soybean products (Toro‐Funes, Bosch‐Fuste, Latorre‐Moratalla, Veciana‐Nogués, & Vidal‐Carou, 2015). The present results showed that putrescine and cadaverine were the most prevalent amines in the two types of sufu. Notably, putrescine and cadaverine associate with unsanitary conditions, suggesting a lack of good hygienic practices during the manufacturing of sufu (Leitão, Marques, & Romão, 2005). The variations in the biogenic amines in different types of sufu are probably due to the manufacturing method and microbiological composition (Lu et al., 2010). Compared with traditionally fermented high‐salt sufu, the content of total biogenic amines was low in low‐salt sufu. The present results suggest that microwave sterilization inactivated the contaminating microorganisms that were introduced by the biogenic amines. Thus, our results not only provide a theoretical basis for the preparation of low‐salt sufu, especially low‐salt sufu, but also provide a theoretical guidance for the study of other traditionally fermented low‐salt foods.

## 5 CONCLUSION

The present study showed that microwave sterilization could reduce the microorganism number and salt content in pehtze, accelerate the sufu maturation time, and decrease the biogenic amine content, which are typical characteristics of Kedong sufu, compared with traditionally fermented high‐salt sufu. The salt content and maturation time of sufu were reduced by 10% and 45 days, respectively. Compared with high‐salt sufu, the contents of free amino acids were high in low‐salt sufu. There was no significant difference in the contents and constitutes of typical flavor components between the two types of sufu. The total biogenic amine contents were reduced by 46%. In summary, it is possible to substitute microwave sterilization for high‐salt in the manufacture of Kedong sufu.

## CONFLICT OF INTEREST

There are no competing financial interests associated with this study.

## ETHICAL STATEMENT

This study does not involve any human or animal testing.
